# GB1a Activates SIRT6 to Regulate Lipid Metabolism in Mouse Primary Hepatocytes

**DOI:** 10.3390/ijms24119540

**Published:** 2023-05-31

**Authors:** Yongzhi Sun, Congmin Zheng, Ting Li, Xinqian He, Fan Yang, Wenfeng Guo, Jianping Song, Yong Gao, Changsheng Deng, Xinan Huang

**Affiliations:** 1 Artemisinin Research Center, Guangzhou University of Chinese Medicine, Guangzhou 510405, China; mugui0510@163.com (Y.S.); hcongminzheng@163.com (C.Z.); tingli910@foxmail.com (T.L.); charilce@foxmail.com (X.H.); flora.yang0718@foxmail.com (F.Y.); guowenfeng@gzucm.edu.cn (W.G.); songjp@gzucm.edu.cn (J.S.); 2 Institute of Science and Technology Park, Guangzhou University of Chinese Medicine, Guangzhou 510405, China; 3 Science and Technology Innovation Center, Guangzhou University of Chinese Medicine, Guangzhou 510405, China; gaoyong@gzucm.edu.cn

**Keywords:** Garcinia biflavonoid 1a, SIRT6, mouse primary hepatocytes, lipid metabolism

## Abstract

Lipid accumulation, oxidative stress, and inflammation in hepatocytes are features of nonalcoholic fatty liver disease (NAFLD). Garcinia biflavonoid 1a (GB1a) is a natural product capable of hepatic protection. In this study, the effect of GB1a on anti-inflammatory, antioxidant, and regulation of the accumulation in HepG2 cells and mouse primary hepatocytes (MPHs) was investigated, and its regulatory mechanism was further explored. The result showed that GB1a reduced triglyceride (TG) content and lipid accumulation by regulating the expression of SREBP-1c and PPARα; GB1a reduced reactive oxygen species (ROS) and improved cellular oxidative stress to protect mitochondrial morphology by regulating genes Nrf2, HO-1, NQO1, and Keap1; and GB1a reduced the damage of hepatocytes by inhibiting the expression of inflammatory cytokines interleukin-6 (IL-6), interleukin-1β (IL-1β), tumor necrosis factor-alpha (TNF-α), and nuclear factor kappa B (NF-κB) p65. The activities of GB1a were lost in liver SIRT6-specific knockout mouse primary hepatocytes (SIRT6-LKO MPHs). This indicated that activating SIRT6 was critical for GB1a to perform its activity, and GB1a acted as an agonist of SIRT6. It was speculated that GB1a may be a potential drug for NAFLD treatment.

## 1. Introduction

Nonalcoholic fatty liver disease (NAFLD) is characterized by lipid accumulation in the liver, which can develop into nonalcoholic steatohepatitis as the disease continues [1,2]. The previous mainstream view was that the first hit to liver cells is the interaction of obesity, a high-fat diet, and bad living habits, which promote the up-regulation of lipid-producing transcription factors and cause lipid accumulation in cells [3], while the second hit is oxidative stress and inflammation. These hits are mutually causal in the course of NAFLD and continue to aggravate the disease [4,5]. With the deepening of research, the ‘multiple hits’ theory has been gradually accepted, which includes insulin resistance, adipose tissue hormone secretion, nutritional factors, intestinal microorganisms, and epigenetic factors that cause various types of obsolete lipid accumulation [6]. The pathological change in NAFLD follows a three-part process: steatosis, lipotoxicity, and inflammation [7]. Therefore, reducing lipid accumulation and relieving early inflammation in hepatocytes may delay the course of NAFLD.

Histone modification is an important component of epigenetics, and histone deacetylases are potential therapeutic targets for several diseases. As a family member, sirtuin6 (SIRT6) is an NAD+-dependent histone deacetyltransferase and is only expressed in the nucleus [8,9,10,11]. Except for removing the acetyl groups from histones, SIRT6 exhibits activities of anti-stress, maintaining genomic stability, anti-aging, and improving inflammation of liver tissue cells [12,13]. Studies have found that when cells lose the protection of SIRT6, excessive lipid accumulation will damage the redox system of cells; stimulate the production of reactive oxygen species (ROS); and attack mitochondria with regard to its permeability, membrane potential, and structural changes, which leads to cell inflammation or apoptosis and promotes NAFLD to continue to develop [14,15].

Currently, there is no approved drug for treating NAFLD, and the symptomatic treatment drug is given based on the course of the disease [2]. Animal studies have confirmed that the extract of *Garcinia cola* has hepatoprotective, antioxidant, anti-free radical, and anti-inflammatory effects, and GB1a is an ingredient in this extract [16,17,18,19,20,21,22,23,24]. Our previous experiment found that GB1a had anti-inflammatory and antioxidant activities in mice with ulcerative colitis [25], and its structural analog GB1 (compared with GB1a, GB1 had hydroxyl substitution at site 3 in flavonoids) could reduce lipid deposition in HepG2 cells via regulating PPARα [26]. With regard to the comprehensive consideration of the activities of GB1 and GB1a, in this paper, we examined the activity of GB1a in improving lipid accumulation, oxidative stress, and inflammation in liver cells and investigated the role of SIRT6 in these activities. 

## 2. Results

### 2.1. The Effect of GB1a on the HepG2 Adipophilin Model

#### 2.1.1. GB1a Decreased OA&PA-Induced Lipid Accumulation in HepG2 Cells

**Figure 1:**
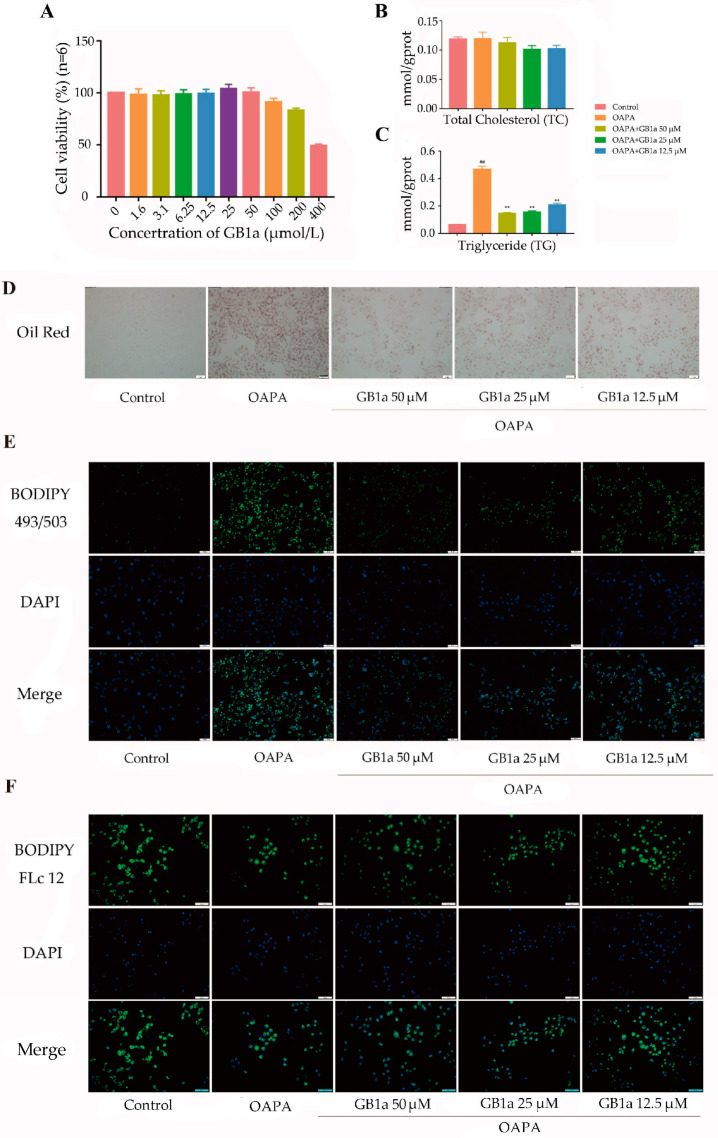

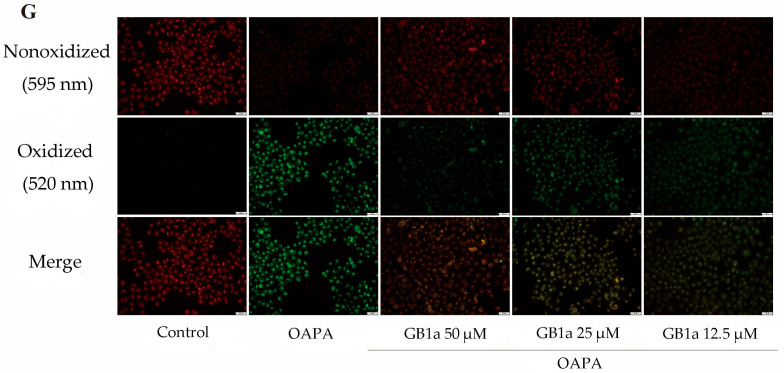
The effect of GB1a on HepG2 adipophilin model. (**A**) HepG2 cells under different concentrations of GB1a, cell activity was assessed with the CCK-8 assay kit, and the colors in this bar are unrelated and represent only different concentrations of GB1a. (**B**,**C**) Triglyceride and total cholesterol levels under different conditions were detected by triglyceride and total cholesterol kit. (**D**) Oil red ‘O’ staining, observed by microscope (200×). (**E**) Lipid accumulation, observed by Bodipy 493/503 (green) fluorescent staining (200×). (**F**) Fatty acid uptake was determined by Bodipy FLc12 fluorescence staining (200×). (**G**) Lipid peroxidation, observed by Bodipy 581/591 (red: unoxidized, green: post-oxidized) fluorescence staining (200×). All data are presented as mean ± SEM of three independent experiments. ^##^, *p* < 0.01 vs. control group; **, *p* < 0.01 vs. OA&PA group.

GB1a showed low cytotoxicity against HepG2 cells under 50 μM (Figure 1A). GB1a significantly decreased intracellular TG levels (Figure 1C). The oil red O staining also showed decreased intracellular lipid levels (Figure 1D). Lipid probe staining showed that lipid accumulation, fatty acid uptake, and lipid peroxidation were improved (Figure 1E–G). The results revealed that GB1a had an effect on reducing lipid accumulation in HepG2 cells. [monounsaturated oleic acid and saturated palmitic acid (OA&PA)].

#### 2.1.2. GB1a Inhibited Lipogenic Genes and Promoted the Expression of Lipid Oxidation Genes in HepG2 Cells

**Figure 2:**
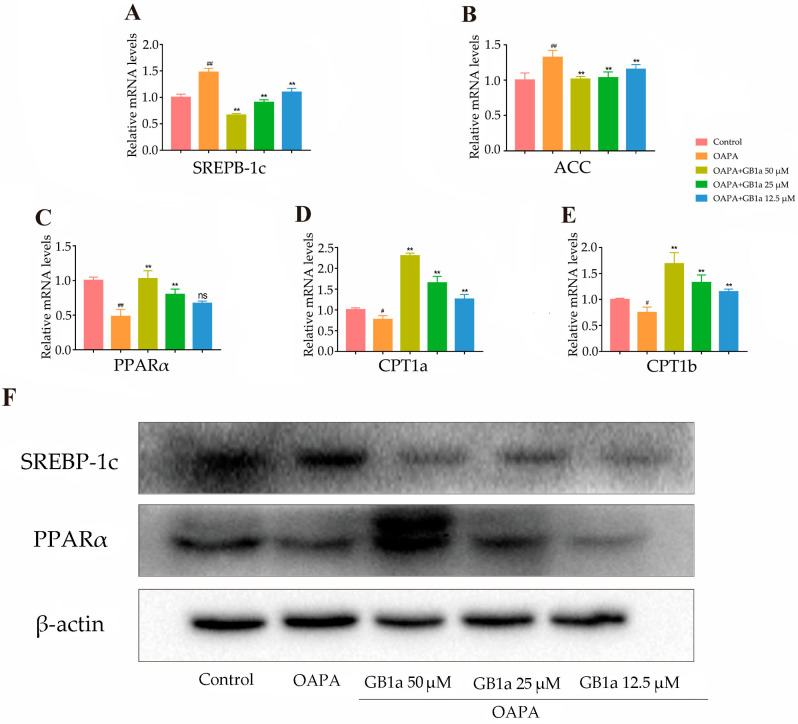
The positive regulation of GB1a on lipid metabolism. (**A**–**E**) GB1a inhibited the lipogenic genes and promoted lipid oxidation gene levels in HepG2 cells; (**F**) the expression level of lipid metabolism protein in cells. All data are presented as mean ± SEM of three independent experiments. ^#^, *p* < 0.05, ^##^, *p* < 0.01 vs. control group; **, *p* < 0.01 vs. OA&PA group; ns, not significant vs. OA&PA group.

The effect of GB1a on lipid metabolism was examined at the molecular level. The results revealed that GB1a inhibited the transcription of ACC, which encodes lipogenesis, and promoted the transcription of CPT1a and CPT1b (Figure 2A–E); meanwhile, GB1a inhibited the expression of liver lipid synthesis protein SREBP-1c and up-regulated the expression of fatty acid oxidation protein PPARα (Figure 2F). Combined with the results of Section 2.1.1, GB1a had a positive effect on lipid metabolism.

#### 2.1.3. GB1a Improved the Oxidative Stress Induced by OA&PA in HepG2 Cells

**Figure 3:**
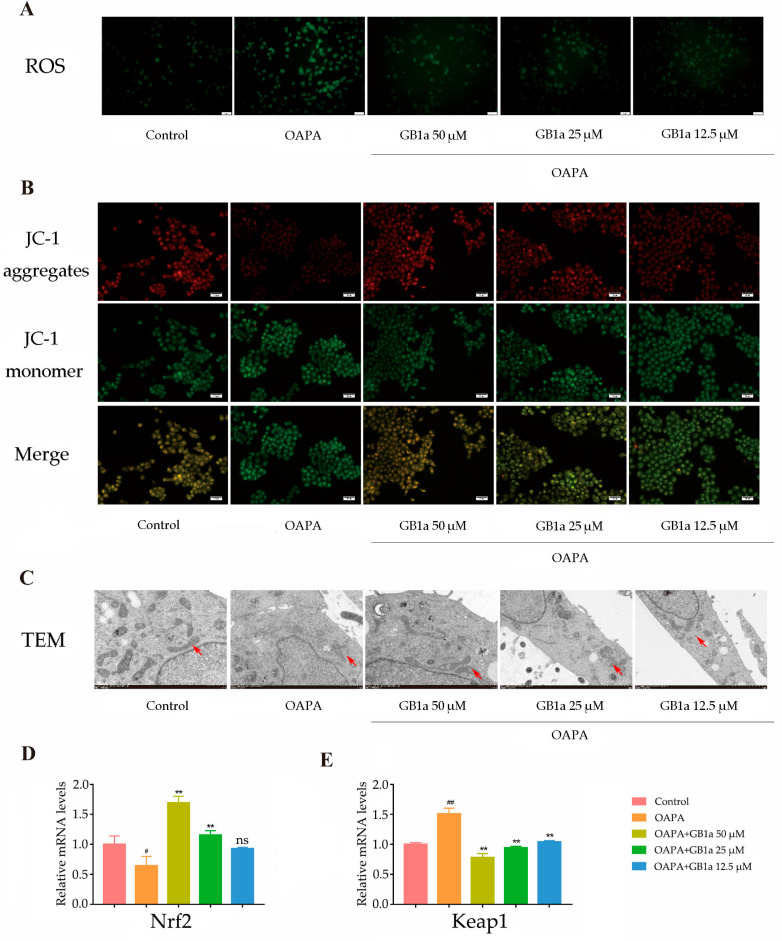

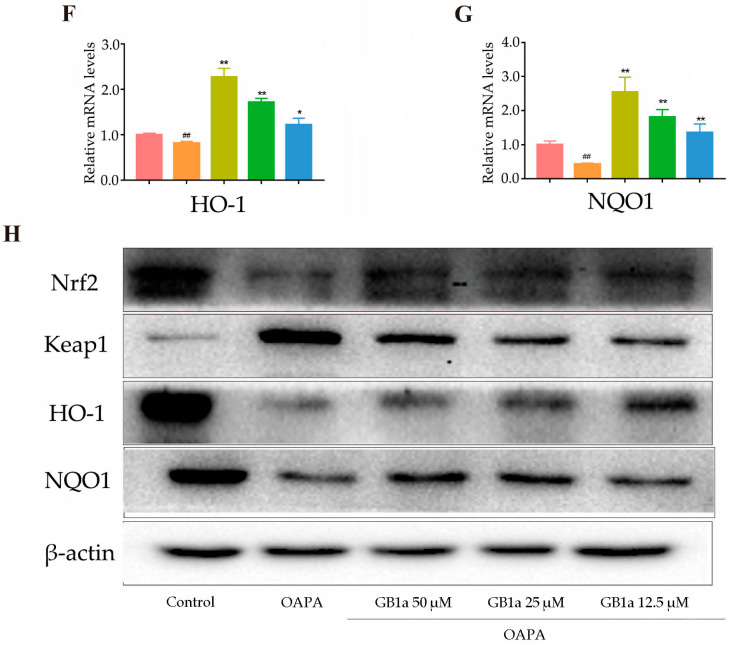
Effects of GB1a on oxidative stress in HepG2 cells. (**A**) The levels of ROS in HepG2 cells, observed by microscope (200×). (**B**) The mitochondrial membrane potential after GB1a treatment under inverted fluorescence microscope (200×). When the mitochondrial membrane potential is high, JC-1 aggregates in the matrix of the mitochondria to form the polymer (J-aggregates), which produces red fluorescence; when the mitochondrial membrane potential is low, JC-1 cannot gather in the matrix of mitochondria, and at this time, JC-1 is a monomer, which produces green fluorescence. (**C**) The mitochondrial morphology of HepG2 cells, observed by transmission electron microscopy (8000×), the red arrow points to the mitochondria in the cell; (**D**–**H**) GB1a promoted the expression of antioxidant genes in OA&PA-cultured HepG2 cells. All data are presented as mean ± SEM of three independent experiments. ^#^, *p* < 0.05, ^##^, *p* < 0.01 vs. control group; *, *p* < 0.05, **, *p* < 0.01 vs. OA&PA group; ns, not significant vs. OA&PA group.

The continuous accumulation of intracellular lipids can cause oxidative stress, which in turn produces excessive ROS, the latter leading to mitochondrial damage. Enhanced green fluorescence was observed microscopically, indicating excessive intracellular ROS production in the OA&PA group (Figure 3A). The results showed that GB1a significantly reduced ROS production, as shown by decreased green fluorescence; GB1a restored mitochondrial membrane potential and showed enhanced red fluorescence; and GB1a also repaired abnormal morphology, such as mitochondrial enlargement (Figure 3A–C). Moreover, GB1a promoted the expression of Nrf2, up-regulated antioxidant genes HO-1 and NQO1 (Figure 3D–H), and reduced cell apoptosis.

#### 2.1.4. GB1a Alleviated the OA&PA-Induced Inflammatory Response in HepG2 Cells

**Figure 4:**
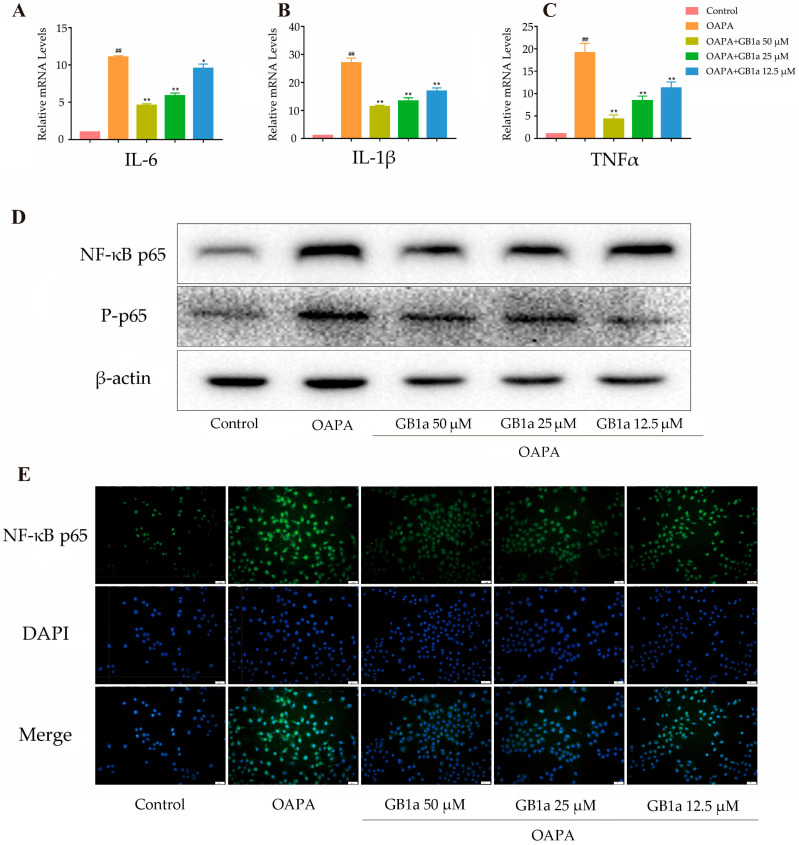
GB1a significantly alleviated inflammation in the HepG2 cell model. (**A**–**D**) The expression of inflammatory factors IL-6, IL-1β, TNF-α, NF-κB, and p-NF-κB in cells after GB1a treatment; (**E**) the expression of NF-κB in HepG2 cells, detected by immunofluorescence (200×). All data are presented as mean ± SEM of three independent experiments. ^##^, *p* < 0.01 vs. control group; *, *p* < 0.05, **, *p* < 0.01 vs. OA&PA group.

Oxidative stress can activate the NF-κB pathway to aggravate inflammation. Our results showed that GB1a reduced the expression of inflammatory cytokines IL-6, IL-1β, and TNF-α by inhibiting the activation of the NF-κB p65 pathway (Figure 4A–E), finally alleviating the inflammatory response.

#### 2.1.5. GB1a Acted as an Agonist of SIRT6

**Figure 5:**
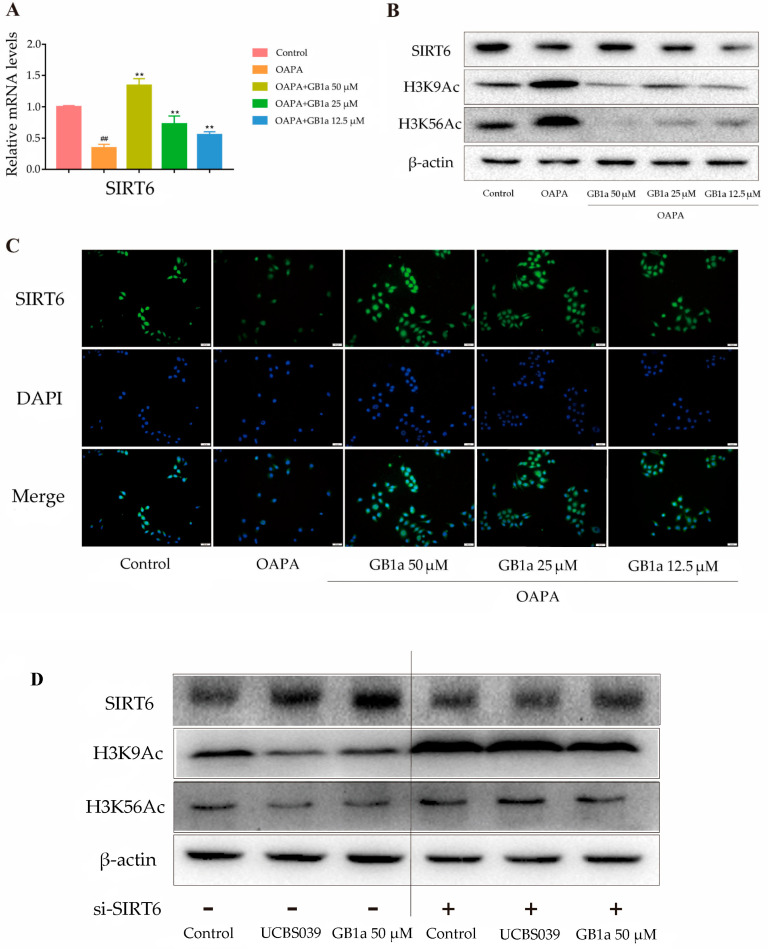
GB1a acted as an agonist of SIRT6. (**A**–**C**) The activation of SIRT6 was accompanied by the deacetylation of H3K9 and H3K56, and Figure 5C was observed by microscope (200×); (**D**) GB1a and UBCS039 affected the expression of SIRT6 in HepG2 cells. All data are presented as mean ± SEM of three independent experiments. ^##^, *p* < 0.01 vs. control group; **, *p* < 0.01 vs. OA&PA group.

GB1a up-regulated the mRNA and protein expression of SIRT6 in HepG2 cells in a dose-dependent manner, while OA&PA decreased their expressions (Figure 5A). GB1a could activate SIRT6 to deacetylate H3K9 and H3K56 and finally reduce the acetylation of H3K9 and H3K56 (Figure 5B). SIRT6 activation by GB1a was also verified by a cell immunofluorescence assay. In this experiment, GB1a restored the expression of SIRT6 inhibited by OA&PA (Figure 5C).

Similar to the SIRT6 agonist UBCS039, GB1a decreased the acetylation levels of H3K9 and H3K56 and promoted the expression of the SIRT6 protein. After silencing the SIRT6 gene, SIRT6 was not expressed in the siRNA-SIRT6 group, siRNA-SIRT6 + UBCS039 group, or siRNA-SIRT6 + GB1a-H group, and the acetylation levels of H3K9 and H3K56 remained unchanged (Figure 5D). Thus, we speculate that GB1a may play a role as an agonist of SIRT6.

#### 2.1.6. GB1a Has Good Combination with SIRT6

**Figure 6:**
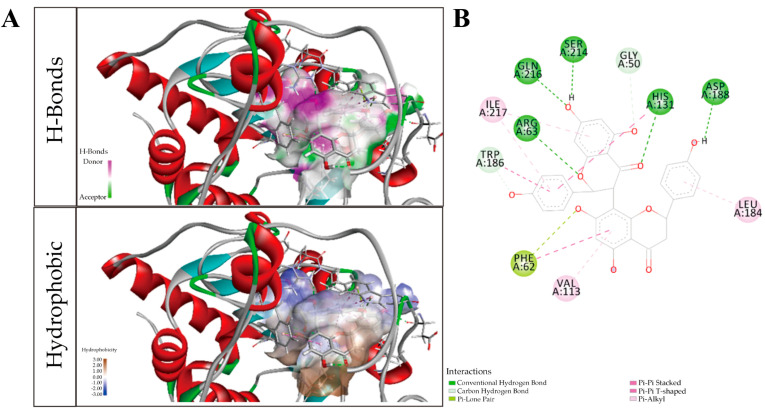
(**A**) The 3D schematic diagram of combined GB1a and SIRT6–RHD; above: hydrogen bond donor and recipient region, pink area indicates the hydrogen bond donor region; green area indicates hydrogen bond recipient region; and the following figure displays the hydrophobic area, with brown representing the hydrophobic area and blue representing the hydrophilic area; (**B**) the detailed 2D binding pattern of GB1a and SIRT6.

The binding force between GB1a and SIRT6 was detected via molecular docking. The result showed that the total score of SIRT6 (5X16) protein docking with GB1a was 12.1993, and the CSCORE was 3 (Table 1). The affinity between 5X16 and GB1a mainly came from their comprehensive interactions. Specifically, GB1a formed hydrogen bonds with the protein residues of GLN 216, SER 214, and ARG 63; carbon–hydrogen bonds with GLY 50 and TRP 186; π lone pair electron interaction with PHE 62; π–π T-type conjugations with PHE 62 and TRP 186; and π-bond hydrophobic interactions with ILE 217, VAL 113, and LEU 184 (Figure 6). Therefore, there was a theoretical possibility that SIRT6 and GB1a could be combined.

### 2.2. Regulation Effect of GB1a on Lipid Metabolism in MPHs

#### 2.2.1. GB1a Failed to Reduce OA&PA-Induced Lipid Accumulation in SIRT6-LKO MPHs

**Figure 7:**
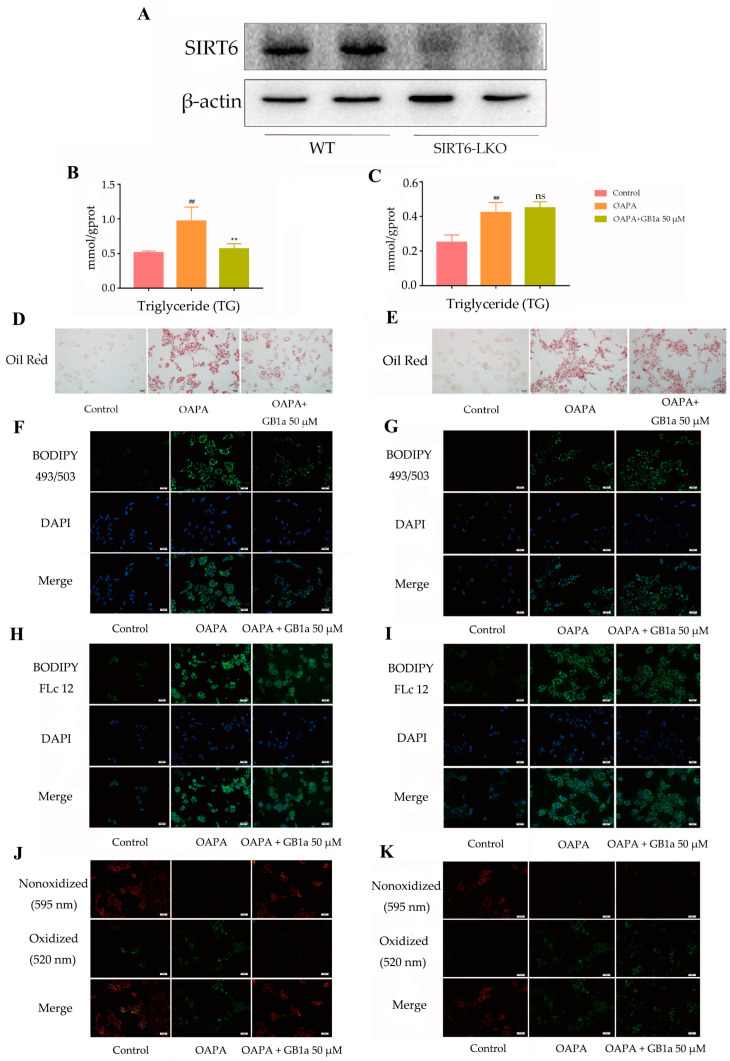

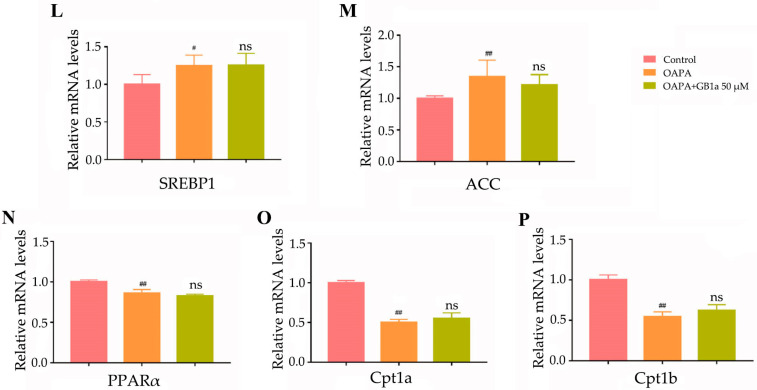
GB1a failed to reduce OA&PA-induced lipid accumulation in SIRT6-LKO MPHs. (**A**) Verification of the effect of SIRT6 knockout; (**B**,**C**) the changes in triglycerides in MPHs and SIRT6-LKO MPHs were detected by the triglyceride kit; (**D**,**E**) the oil red O staining of MPHs and SIRT6-LKO MPHs was observed by microscope (200×); (**F**,**G**) lipid accumulation in MPHs and SIRT6-LKO MPHs was observed by Bodipy 493/503 (green) fluorescent staining (200×); (**H**,**I**) fatty acid uptake in MPHs and SIRT6-LKO MPHs was determined by Bodipy FLc12 fluorescence staining (200×); (**J**,**K**) lipid peroxidation in MPHs and SIRT6-LKO MPHs was observed by Bodipy 581/591 (red: unoxidized, green: post-oxidized) fluorescence staining (200×). (**L**–**P**) The level of lipid metabolism genes in SIRT6-LKO MPHs after GB1a treatment. All data are presented as mean ± SEM of three independent experiments. ^#^, *p* < 0.05, ^##^, *p* < 0.01 vs. control group; **, *p* < 0.01 vs. OA&PA group; ns, not significant vs. OA&PA group.

The MPHs from C57BL/6 mice were used to verify whether GB1a could reduce OA&PA-induced lipid accumulation. We first verified the knockout effect of SIRT6 in MPHs via Western blotting and found that no SIRT6 protein was detected on this strip (Figure 7A), which indicated that the knockout was successful. After GB1a intervention (50 μM), MPHs’ intracellular TG content decreased (Figure 7B), and oil red O staining also showed a decrease in lipid level (Figure 7D). Moreover, the lipid probe staining showed that lipid accumulation, fatty acid uptake, and lipid peroxidation were alleviated in GB1a-treated cells (Figure 7F,H,J). However, with the knockout of SIRT6 in MPHs, GB1a’s role in the above regulation of lipid metabolism was eliminated (Figure 7C,E,G,I,K). Compared with the results of 2.1.2, the genes related to lipid metabolism were not altered in SIRT6-LKO MPHs.

#### 2.2.2. GB1a Lost Protection against Oxidative Stress and the Inflammatory Response Due to OA&PA in SIRT6-LKO MPHs

**Figure 8:**
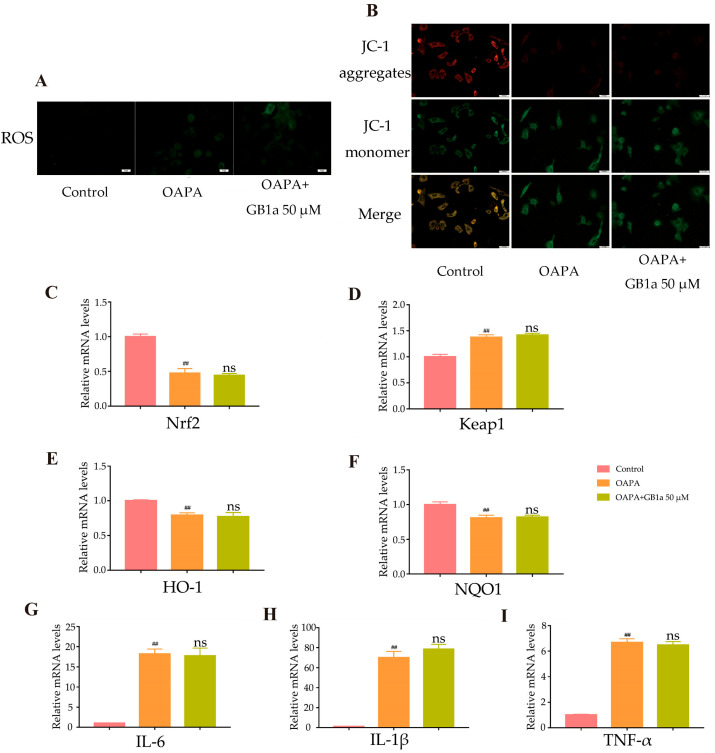
GB1a failed to improve OA&PA-induced oxidative stress and inflammation in SIRT6-LKO MPHs. (**A**,**B**) The intracellular ROS and mitochondrial membrane potential in SIRT6-LKO MPHs after GB1a treatment under an inverted fluorescence microscope (200×); (**C**–**I**) the level of anti-inflammatory and antioxidant genes in SIRT6-LKO MPHs after GB1a treatment. All data are presented as mean ± SEM of three independent experiments. ^##^, *p* < 0.01 vs. control group; ns, not significant vs. OA&PA group.

Finally, we found that GB1a could not exert anti-inflammatory and antioxidant effects in SIRT6-LKO MPHs (Figure 8A,B), and the levels of antioxidant genes and inflammatory factors were not improved (Figure 8C–I).

## 3. Discussion

NAFLD is a metabolic-related disease. Studies have shown that significant lipid accumulation in hepatocytes indicates the progression of the disease, so maintaining liver lipid metabolism homeostasis is essential for the prevention and treatment of NAFLD [27]. The most abundant fatty acids in people’s daily diets are saturated PA and monounsaturated OA. OA is prone to lipid accumulation, and PA is more likely to induce oxidative stress [28]. Long-term lipid exposure and excessive lipid accumulation in cells seriously damage the redox system, destroy mitochondrial biogenesis, and subsequently lead to inflammation or apoptosis, which ultimately promotes the development of NAFLD [29,30,31,32].

SIRT6 is reported to be involved in the regulation of various metabolic processes, including glycolysis, gluconeogenesis, liver lipids, and cholesterol [13,33,34,35,36,37,38]. In mice with systemic knockout of SIRT6, aging phenotypes and severe inflammatory responses occur. The specific knockout of SIRT6 in T cells or macrophages causes the attack of immune cells and the excessive secretion of inflammatory factors, which ultimately promotes the occurrence of liver fibrosis [13]. After a high-fat diet, liver-specific SIRT6 knockout mice have increased glycolysis, de novo lipogenesis, and β-oxidation, which aggravate fatty liver and insulin resistance [36,39]. The above research shows that the activation of SIRT6 can effectively inhibit intracellular lipid accumulation and reduce the damage caused by inflammation and oxidative stress.

In our study, GB1a could promote the expression of SIRT6 and reduce the acetylation levels of H3K9 and H3K56, just like the SIRT6 agonist UBCS039 in HepG2 cells, which was further verified in OA&PA-induced MPHs. Moreover, we found that GB1a lost its capability of regulating the expressions of lipid-metabolism-related genes and did not improve ROS and mitochondrial membrane potential in the OA&PA-inducted SIRT6-LKO MPHs. These results showed that activating SIRT6 was critical for GB1a. Since the molecular docking result also supported the binding of SIRT6 and GB1a, we speculated that SIRT6 might be the protein target of GB1a. In addition, our research group will extract and separate GB1a from another batch of *Garcinia cola* fruit for in vivo experiments in NALFD animal models so as to enrich the pharmacological research on GB1a regulating lipid metabolism by activating SIRT6.

## 4. Materials and Methods

### 4.1. The Source of GB1a and Cells

GB1a was purified from *Garcinia cola* fruit, as described in [25].

HepG2 cells were obtained from the Shanghai Cell Bank of the China Academy of Sciences (Shanghai, China). MPHs were isolated from C57BL/6 mice, male, weighing about 21–24 g, and 6 weeks old, purchased from Guangdong Medical Laboratory Animal Center, Certificate No. SCXK (Guangzhou, China) 2018-0002.

### 4.2. Cell Viability and GB1a Administration

Cells were inoculated in a 96-well plate at 5 × 10^3^ cells/well overnight. On the following day, the cells were exposed to a mixture of free fatty acids (saturated palmitic acid (PA)/monounsaturated oleic acid (OA) = 2:1) and then treated with GB1a for 24 h, compared with those not treated with GB1a as a control. Cell activity was assessed with the CCK-8 assay kit.

### 4.3. Western Blot

The cells were cultured and treated in a Petri dish (60 mm). After removal, the medium was discarded and washed twice with PBS. The total protein was extracted, and the same amount of protein was separated by electrophoresis and transferred to a polyvinylidene fluoride (PVDF) membrane (Millipore Corporation 290 Concord Road Billerica, MA 01821). Then, we blocked the membrane with 5% skim milk, followed by incubation with anti-SIRT6 (R1511-1, HUABIO, Hangzhou, China), anti-PPARα (bs-23398R, Bioss, Beijing, China), acetyl-histone H3K56 antibody (A7256, ABclonal, Wuhan, China), anti-NF-κB p65 (ab86299, Abcam, Hangzhou, China), and anti-β-actin (EM21002, HUABIO, Hangzhou, China). Relative expression of the proteins was normalized to the actin protein. The total protein load was 60 µg/µL. The dilution ratio of primary antibody was 1:1000, and that of secondary antibody was 1:5000. The type of secondary antibody was goat anti-rabbit. Chemiluminescence signals were quantified using a chemiluminescence imaging system (BIO-RAD).

### 4.4. Real-Time Quantitative Polymerase Chain Reaction (RT-qPCR)

Cells were harvested to extract total RNA using Trizol reagent, following culture in a 6-well plate and PBS washing two times. Reverse transcription was conducted using RT-MIX to obtain cDNA. RT-qPCR amplification was performed with the SYBR Green Master Mix (Thermo Fisher Scientific, Waltham, MA, USA). RT-qPCR cycle conditions: 95 °C, 1 min predenaturation; 95 °C, 5 s denaturation; 60 °C, 30 s annealing, extension, and 40 cycles. Detailed primer sequences are listed in Table 2. RNA expression level was normalized to actin.

### 4.5. Isolation and Culture of Mouse Primary Hepatocytes

(1) We spread 0.15% gelatin on the culture plate or Petri dish, shook it well, and placed it for 3–5 min, then absorbed and discarded the gelatin and sterilized it with ultraviolet light for 30 min; (2) mice were anesthetized with pentobarbital sodium. The inferior vena cava was separated, and a catheter was placed at the distal end of the inferior vena cava. A total of 1–2 mL of heparin was injected immediately after blood return. After heparin injection, 50 mL of perfusion fluid Ⅰ was inserted; (3) during this period, 0.015 g of collagenase was dissolved in 30 mL of perfusion solution Ⅱ, mixed, and kept at 37 °C; (4) we irrigated lavage solution Ⅱ at a slow speed for about 6 min until the liver was soft and chapped, and then stopped lavage; (5) the whole liver was removed and placed in a Petri dish, and then transferred to an ultra-clean platform with 20 mL of basic medium. The liver was torn, 70 µm of cell network was filtered, and the filtrate was collected. We centrifuged at 800 rpm/min for 3 min and discarded the supernatant. We repeated twice; (6) we added RPMI complete medium. A trypan blue count of living cells (more than 80% of living cells can continue the follow-up experiment) was performed; (7) 100% of cells were inoculated, the fluid was changed 2 h later, and the experiment could be started after 12–24 h when cells were observed to be fully adherent to the wall.

### 4.6. Lipid Probe Staining

Lipid accumulation, fatty acid uptake, and lipid peroxidation were examined using the fluorescent lipid probes BODIPY^TM^ 493/503, BODIPY^TM^ FCL12, and BODIPY^TM^ 581/591 (ThermFisher Scientific, Waltham, MA, USA). According to the manufacturer’s instructions, the cells were, respectively, incubated with lipid and fatty acid probes (10 µ/mL) for 20 min in the dark. The cells were washed with buffer solution, and then observations were made using an inverted fluorescence microscope.

### 4.7. Cellular Immunofluorescence

After group treatment, the cells were washed with pre-cooled PBS 3 times. We fixed it with 4% paraformaldehyde, placed it at 4 °C for 30 min, and washed it with PBS for 10 min. In the form of 0.5% Triton X-100 (PBS), it was permeated at room temperature for 20 min. Then, a 5% BSA solution was used as the sealing solution, sealed for 1 h, and the sealing solution was abandoned. The proportionally diluted primary antibody (R1511-1, SIRT6, HUABIO, Hangzhou, China) was added, the cell tablet was covered with liquid, and the antibody was recovered and washed with PBS for 10 min after 60 rotations and 4 °C overnight in the shaking bed. The FITC-labeled fluorescent secondary antibody (CW0103, goat anti-rabbit IgG, CWBIO, Beijing, China) was diluted at 1:200 and added to 24-well plates. It was appropriate to cover the cells with liquid. After incubation for 1 h at room temperature and away from light, the antibody was recovered and washed with PBS for 10 min. The above operation was repeated three times. We drained the water on the slide, dropped 6 μL of anti-quenching fluorescent tablet containing DAPI onto the slide, and placed the slide upside down. The nail polish was fixed and observed with a fluorescence microscope.

### 4.8. Detection of Reactive Oxygen Species (ROS)

The ROS was measured via the fluorescent probe DCFH-DA using the Reactive Oxygen Species Assay Kit (S0033S, Beyotime, Shanghai, China). Briefly, the pretreated cells were washed twice with PBS, a diluted DCFH-DA probe was added, and then they were incubated at 4 °C in the dark for 20 min. After washing and attaching it to the slide, the ROS was observed under a fluorescence microscope.

### 4.9. Detection of Mitochondrial Membrane Potential (MMP)

The MMP was measured by JC-1 dye using the JC-1 (C006, Beyotime, Shanghai, China) Mitochondrial Membrane Potential Assay Kit. Briefly, the pretreated cells were washed twice with PBS, a pre-cooled JC-1 working solution was applied to stain the cells, and then they were incubated at 4 °C in the dark for 30 min. After washing and attaching it to the slide, the MMP was observed under a fluorescence microscope.

### 4.10. The morphology and Structure of Cell Mitochondria Were Observed by Transmission Electron Microscopy

The pretreated cells were washed with pre-cooled PBS 3 times. We added 1 mL of room temperature 2.5% butanediol fixing solution to each well and fixed it at room temperature for 5 min away from light; the cells were then gently scraped in one direction with a cell scraper, and the cell suspension was transferred to a 1.5 mL enzyme-free EP tube under centrifugation conditions of 3000 rpm for 5 min. After centrifugation, fixation, dehydration, embedding, and solidification were carried out according to the following steps: 2.5% glutaraldehyde for 2 h, 1 M PBS Buffer (PH 7.2) for 15 min, repeated 3 times; 1% osmic acid for 2.5 h, 1 M PBS buffer (PH 7.2) for 15 min, repeated 3 times; and 30%, 50%, 70%, 90%, and 100% acetone for 30 min, 4 °C. Anhydrous acetone/embedding agent (2:1) 4 h, anhydrous acetone/embedding agent (1:2) overnight, embedding agent 3 h, 37 °C, 45 °C over 12 h, 60 °C oven 48 h. The sections were sliced via an ultra-thin microtome, and the section area was no more than 0.5 × 0.3 mm. Finally, the staining was finished by washing with lead citrate for 10 min and ddH2O without carbon dioxide 3 times, and uranium acetate for 30 min and ddH2O without carbon dioxide 3 times. We let it dry naturally and observed it via transmission electron microscopy.

### 4.11. Molecular Docking

We downloaded the GB1a structure from the PubChem database and optimized it to obtain the most stable structure. The three-dimensional crystal structure of the SIRT6 protein was downloaded from the RCSB PDB database, and the protein structure was corrected by hydrogenation, side chain repair, charge addition, extraction of inlay ligand, and other operations. Total score and C–score were used to evaluate the molecular docking effect. The total score function comprehensively considered molecular polarity, hydrophobicity, enthalpy, solvation, and other factors. The molecular surface properties were calculated by MOLCAD, and the binding mode and interaction mechanism between GB1a and SIRT6 were analyzed. All calculations were performed using SYBYL–X 2.2.1 software, and the resulting demonstration was performed using Discover Studio 2016 software.

### 4.12. Statistical Analysis

The experimental data were statistically analyzed using SPSS 26.0 statistical analysis software and expressed as mean ± standard deviation ($\bar{X}$ ± s). The differences between the measurement data groups conforming to the normal distribution (Shapiro–Wilk test, *p* > 0.05) were tested by the one-way ANOVA method. The comparison between groups was conducted in pairs. The homogeneity of variances was tested by the LSD method, and the heterogeneity was tested by Dunnett’s t3 method. * *p* < 0.05 indicated a statistically significant difference.

## 5. Conclusions

In summary, our study revealed that GB1a reduced lipid accumulation and inflammatory response and improved oxidative stress in OA&PA-induced adipophilin cell models. SIRT6 is essential for these pharmacological activities of GB1a described above, and GB1a could act as an agonist of SIRT6 to delay the pathological progression of NAFLD.

## Figures and Tables

**Table 1:** The docking result of GB1a and 5X16.

Chemical	Resolution	PDB_ID	Total Score	Crash	Polar	C_SCORE
GB1a	1.97Å	5X16	12.1993	−3.8572	4.2729	3

**Table 2:** Primers used for real-time qRT-PCR. (Human (h), mouse (m)).

Primer	Sequences
SIRT6 (h)	F1:5′-CCTCTCTCTAATCAGCCCTCTG-3′
	R1:5′-GAGGACCTGGGAGTAGATGAG-3′
SIRT6 (m)	F1:5′-ATGTCGGTGAATTATGCAGCA-3′
SREBP-1c (h)	R1:5′-GCTGGAGGACTGCCACATTA-3′ F1:5′-ACAGTGACTTCCCTGGCCTAT-3′ R1:5′-GCATGGACGGGTACATCTTCAA-3′
SREBP-1c (m)	F1:5′-TGACCCGGCTATTCCGTGA-3′
	R1:5′-CTGGGCTGAGCAATACAGTTC-3′
PPARα (m)	F1:5′-AGAGCCCCATCTGTCCTCTC-3′
ACC (h)	R1:5′-ACTGGTAGTCTGCAAAACCAAA-3′ F1:5′-ATGTCTGGCTTGCACCTAGTA-3′ R1:5′-CCCCAAAGCGAGTAACAAATTCT-3′
ACC (m)	F1:5′-CTTGGGTGCTGACTACAACC-3′
Cpt1a (h)	R1:5′-GCCCTCCCGTACACTCACTC-3′ F1:5′-TCCAGTTGGCTTATCGTGGTG-3′ R1:5′-TCCAGAGTCCGATTGATTTTTGC-3′
Cpt1a (m)	F1:5′-CTCCGCCTGAGCCATGAAG-3′
Cpt1b (h)	R1:5′-CACCAGTGATGATGCCATTCT-3′ F1:5′-GCGCCCCTTGTTGGATGAT-3′ R1:5′-CCACCATGACTTGAGCACCAG-3′
Cpt1b (m)	F1:5′-GCACACCAGGCAGTAGCTTT-3′
TNF-α (h)	R1:5′-CAGGAGTTGATTCCAGACAGGTA-3′ F1:5′-CCTCTCTCTAATCAGCCCTCTG-3′ R1:5′-GAGGACCTGGGAGTAGATGAG-3′
TNF-α (m)	F1:5′-CCCTCACACTCAGATCATCTTCT-3′
IL-1β (h)	R1:5′-GCTACGACGTGGGCTACAG-3′ F1:5′-ATGATGGCTTATTACAGTGGCAA-3′ R1:5′-GTCGGAGATTCGTAGCTGGA-3′
IL-1β (m)	F1:5′-GCAACTGTTCCTGAACTCAACT-3′
IL-6 (h)	R1:5′-ATCTTTTGGGGTCCGTCAACT-3′ F1:5′-ACTCACCTCTTCAGAACGAATTG-3′ R1:5′-CCATCTTTGGAAGGTTCAGGTTG-3′
IL-6 (m)	F1:5′-TAGTCCTTCCTACCCCAATTTCC-3′
Nrf2 (h)	R1:5′-TTGGTCCTTAGCCACTCCTTC-3′ F1:5′-TCAGCGACGGAAAGAGTATGA-3′ R1:5′-CCACTGGTTTCTGACTGGATGT-3′
Nrf2 (m)	F1:5′-TCTTGGAGTAAGTCGAGAAGTGT-3′
Keap1 (h)	R1:5′-GTTGAAACTGAGCGAAAAAGGC-3′ F1:5′-CTGGAGGATCATACCAAGCAGG-3′ R1:5′-GGATACCCTCAATGGACACCAC-3′
Keap1 (m)	F1:5′-TGCCCCTGTGGTCAAAGTG-3′
HO-1 (h)	R1:5′-GGTTCGGTTACCGTCCTGC-3′ F1:5′-AAGACTGCGTTCCTGCTCAAC-3′ R1:5′-AAAGCCCTACAGCAACTGTCG-3′
HO-1 (m)	F1:5′-AAGCCGAGAATGCTGAGTTCA-3′
NQO1 (h)	R1:5′-GCCGTGTAGATATGGTACAAGGA-3′ F1:5′-GAAGAGCACTGATCGTACTGGC-3′ R1:5′-GGATACTGAAAGTTCGCAGGG-3′
NQO1 (m)	F1:5′-AGGATGGGAGGTACTCGAATC-3′
	R1:5′-AGGCGTCCTTCCTTATATGCTA-3′
β-actin (m)	F1:5′-GGCTGTATTCCCCTCCATCG-3′
	R1:5′-CCAGTTGGTAACAATGCCATGT-3′
β-actin (h)	F1:5′-CATGTACGTTGCTATCCAGGC-3′
	R1:5′-CTCCTTAATGTCACGCACGAT-3′

## Data Availability

All data generated or analyzed during this study are included in this article.
